# Bioactive flavonoid *p*-hydroxycinnamic acid stimulates osteoblastogenesis and suppresses adipogenesis in bone marrow culture

**DOI:** 10.1007/s00441-013-1707-6

**Published:** 2013-09-13

**Authors:** Masayoshi Yamaguchi, Clifton A. Baile, Shijun Zhu, Mamoru Shoji

**Affiliations:** 1 Department of Hematology and Biomedical Oncology, Winship Cancer Institute, Emory University School of Medicine, Atlanta, GA 30322 USA; 2 Department of Foods and Nutrition, The University of Georgia, Athens, GA 30602 USA; 3 Department of Hematology and Medical Oncology, Winship Cancer Institute, Emory University School of Medicine, 1365 C Clifton Road NE, Atlanta, GA 30322 USA

**Keywords:** *p*-Hydroxycinnamic acid, Osteoblastogenesis, Adipogenesis, 3T3-L1 preadipocytes, Insulin

## Abstract

The bioactive flavonoid *p*-hydroxycinnamic acid (HCA), which is an intermediate-metabolic substance in plants and fruits, is synthesized from tyrosine. The biological effect of HCA is poorly understood. Among cinnamic acid and its related compounds, HCA has a specific-anabolic effect on bone, being found to stimulate osteoblastogenesis and to inhibit osteoclastogenesis through the suppression of NF-κB signaling, thereby preventing bone loss. Bone marrow mesenchymal stem cells give rise to ostoblasts and adipocytes. HCA might therefore have effects on osteoblastogenesis and adipogenesis in bone marrow culture. This study demonstrates (1) that HCA has stimulatory effects on osteoblastogenesis and mineralization and suppressive effects on adipogenesis in mouse bone marrow culture and (2) that HCA depresses adipogenesis in mouse 3T3-L1 preadipocytes in vitro. Such effects of HCA might be involved in the differentiation of mesenchymal stem cells.

## Introduction

Bone homeostasis is maintained through a delicate balance between osteoblastic bone formation and osteoclastic bone resorption. Numerous pathological processes have the capacity to disrupt this equilibrium leading to conditions in which the rate of bone resorption outpaces the rate of bone formation leading to osteoporosis (Weitzmann and Pacifici 2006). The most dramatic expression of osteoporosis is represented by fractures of the proximal femur for which the number increases as the population ages (Johnell and Kanis 2006). Osteoporosis is widely recognized as a major public health threat.

Obesity is currently a major health problem worldwide and is growing in prevalence. Osteoporosis and obesity are now thought to be closely related and to share several features (Gharibi et al. 2011; Rosen and Bouxsein 2006; Kawai and Rosen 2010). One of these shared features is that osteoblasts and adipocytes differentiate from a common precursor cell in the bone marrow, the mesenchymal stem cell. The pluripotency of mesenchymal stem cells is well known and their ability to differentiate into osteoblasts, adipocytes, chondrocytes and myoblasts has been described extensively (Minguell et al. 2001). An inverse relationship exists between the differentiation of mesenchymal stem cells into osteoblasts and adipocytes. Secondary causes of osteoporosis including obesity and diabetes are associated with bone marrow adiposity (Gharibi et al. 2011).

Functional food factors might be important in the prevention and treatment of osteoporosis and obesity. Botanical chemical factors that have an effect on osteoblastogenesis and adipogenesis are poorly understood. Cinnamic acid, a flavonoid, is present in many plants and fruits. The flavonoid *p*-hydroxycinnamic acid (HCA) is an intermediate-metabolic substance in plants and fruits and is synthesized from tyrosine. Among cinnamic acid and its related compounds (cinnamic acid, HCA, ferulic acid, caffeic acid and 3,4-dimethoxycinnamic acid), HCA has been shown to have specific-anabolic effects on bone in vitro (Lai and Yamaguchi 2006a). HCA has been found to stimulate osteoblastic bone formation and to inhibit osteoclastic bone resorption in vitro (Lai and Yamaguchi 2007a; Yamaguchi et al. 2008a) and in vivo (Lai and Yamaguchi 2006b, 2007b; Yamaguchi et al. 2008b), thereby increasing bone mass. HCA has also been found to stimulate osteoblastogenesis and to inhibit osteoclastogenesis through the suppressing of nuclear factor kappa B (NF-κB) signaling, which is activated by tumor necrosis factor-alpha (TNF-α) or the receptor activator of NF-κB ligand (RANKL; Yamaguchi and Weitzmann 2009, 2012), suggesting a molecular mechanism by which HCA has an anabolic effect on bone.

Moreover, HCA has been demonstrated to have preventive effects on ovariectomy-induced bone loss in rats (Yamaguchi et al. 2008b) and the compound has restorative effects on the diabetic state and diabetic-induced bone loss in streptozotocin-induced type I diabetic rats in vivo (Lai and Yamaguchi 2007b). Oral administration of HCA in streptozotocin-induced diabetic rats has also been shown to reduce serum triglyceride and glucose concentrations, which are markedly elevated in the diabetic state in vivo, suggesting that HCA has an effect on lipid metabolism (Lai and Yamaguchi 2007b).

Bone marrow mesenchymal stem cells are multipotent cells, which among other cell lineages, give rise to adipocytes and osteoblasts (Muruganandan et al. 2009). This occurs through cross talk between complex signaling pathways including those derived from bone morphogenic proteins, winglesstype MMTV integration site (Wnt) proteins, the hedgehog proteins, delta/jagged proteins, fibroblastic growth factors, insulin, insulin-like growth factors and transcriptional regulators of adipocyte and osteoblast differentiation including peroxisome proliferators-activated receptor-gamma (PPARγ) and runt-related transcription factor 2 (Runx2; Muruganandan et al. 2009; Laudes 2011). Recently, dietary-induced serum phenolic acids including HCA have been shown to promote bone growth via p38 mitogen-activated protein kinase (MAPK)/beta-catenin canonical Wnt (Chen et al. 2010).

HCA might have effects on the differentiation of bone marrow mesenchymal stem cells and adipogenesis, which is related to differentiation of bone marrow mesenchymal stem cells to adipocytes. The effects of HCA on osteoblastogenesis and adipogenesis in bone marrow culture have not as yet been determined. This study has been undertaken to determine the effects of HCA on osteoblastogenesis and adipogenesis in mouse bone marrow culture and on adipogenesis in mouse 3T3-L1 preadipocytes in vitro. We have found that HCA has stimulatory effects on osteoblastogenesis and suppressive effects on adipogenesis.

## Materials and methods

### Materials

Dulbecco’s modified Eagle’s medium (DMEM) and antibiotics (penicillin and streptomycin) were purchased from Invitrogen (Carlsbad, Calif., USA). Fetal bovine serum (FBS) was from Hyclone. HCA (100 % pure) was obtained from Wako Pure Chemical (Osaka, Japan) and was dissolved in ethanol for use in experiments. Insulin, dexamethasone, 3-isobutyl-1-methylxanthine (IBMX), PD98059, staurosporine and other reagents were purchased from Sigma (St. Louis, Mo., USA). Insulin was dissolved in diluted acidic acid solution and other reagents were dissolved in 100 % ethanol.

### Mineralization in mouse bone marrow culture

Bone marrow cells (1 × 10^6^ cells/wells per 1 ml), which were isolated from mice (C57BL6 wild-type; female, 2 months old) purchased from the Jackson Laboratory (USA), were cultured in 12-well plates in DMEM containing 10 % FBS, 1 % penicillin-streptomycin (P/S; 10,000 U/l) and mineralization medium (MM) containing ascorbic acid (100 ng/ml) and 4 mM β-glycerophosphate in the presence of either vehicle or HCA (1–1000 nM; Yamaguchi et al. 2012). Cells were cultured for 21 days at 37 °C in a humidified 5 % CO_2_ atmosphere. In other experiments, the cells were cultured for 7 days in the presence of HCA (1–1000 nM) or its absence, after which the medium was replaced by medium without HCA and the cells were cultured for an additional 14 days. The medium was changed every 3 days. After culture, the cells were washed with phosphate-buffered saline and stained with Alizarin red stain. For quantitation, 10 % cetylpyridinium chloride solution was added to each well to elute the dye. After complete elution, absorbance of the eluted solution was measured at 570 nm on a microtiter plate reader.

### Adipogenesis in bone marrow culture

Bone marrow cells (1 × 10^6^ cells/well per 1 ml in 12-well plates), which were obtained from the femoral tissues of wild-type mice (female, 3 months old), were cultured for 48 h in DMEM (10 % FBS and 1 % P/S) containing either vehicle or HCA (1–1000 nM) in the presence or absence of the differentiation medium (DM) consisting of dexamethasone (1 μM/ml medium) and IBMX (0.5 mM/ml medium; Yamaguchi et al. 2012). The medium was replaced with α-MEM (with 10 % FBS and 1 % P/S) containing insulin (10 μg/ml medium) without dexamethasone and IBMX and the cells were cultured in the presence of HCA (1–1000 nM) or its absence for 4 days in a CO_2_ incubator (37 °C). In other experiments, the cells were cultured in DM for 48 h with HCA (1–1000 nM) or without HCA and then the medium was replaced and the cells were cultured in medium containing insulin (10 μg/ml medium) for 4 days without HCA. After culture, the medium was removed and the adipocytes were stained with Oil Red O. Adipocytes were counted by light microscopy. For quantification, the dye was extracted with 0.2 ml isopropanol for 1 min and the absorbance (490 nm) was read by using a Spectra Count microplate photometer.

### Culture of 3T3-L1 preadipocytes

3T3-L1 mouse embryo fibroblasts were obtained from the American Type Culture Collection and were cultured as described elsewhere (Hemati et al. 1997). The cells were stored in liquid nitrogen and were thawed in a 37 °C water bath on the day of culture. Cells were then maintained in a 37 °C/5 % CO_2_ humidified environment. Briefly, 3T3-L1 preadipocytes (5000 cells/well per 0.2 ml) were cultured in DMEM containing 10 % bovine calf serum and 1 % P/S in 96-well plates in order to obtain subconfluent undifferentiated cells. After preculture (day 0), the undifferentiated cells were cultured in DM consisting of DMEM (with 10 % FBS and 1 % P/S) containing 0.5 mM IBMX and 1 μM dexamethasone for 2 days and then DM was replaced with 10 % DMEM containing insulin (5 μg/ml) with HCA (0.1–10 μM) or without HCA and the cells were cultured for 2 additional days.

### Lipid assay

Lipid (triglyceride) content in the cells after cell culture was quantified by using the commercially available AdipoRed assay reagent according to the manufacturer’s instructions (Louza, Walkersville, Md., USA; Hemati et al. 1997; Rayalam et al. 2007). Fluorescence was measured at 485–570 nm in a 96-well plate reader (μQuant, Bio-Tek Instruments).

### Cell viability assay

Cell viability assay (CellTiter 96 Aqueous One Solution Cell Proliferation Assay; Promega, Madison, Wis., USA) was performed according to the manufacturer’s instructions. Fluorescence was measured at 560–590 nm in a 96-well plate reader (μQuant, Bio-Tek Instruments) to determine the formazan concentration, which is proportional to the number of cells (Hemati et al. 1997; Rayalam et al. 2007).

### Statistical analysis

Statistical significance was determined by using GraphPad InStat version 3 for Windows XP (GraphPad Software, La Jolla, Calif., USA). Multiple comparisons were performed by one-way analysis of variance (ANOVA) with the Tukey-Kramer multiple comparisons post test for parametric data as indicated. *P* < 0.05 was considered statistically significant.

## Results

### HCA stimulates osteoblastogenesis and mineralization in bone marrow culture

**Fig. 1:**
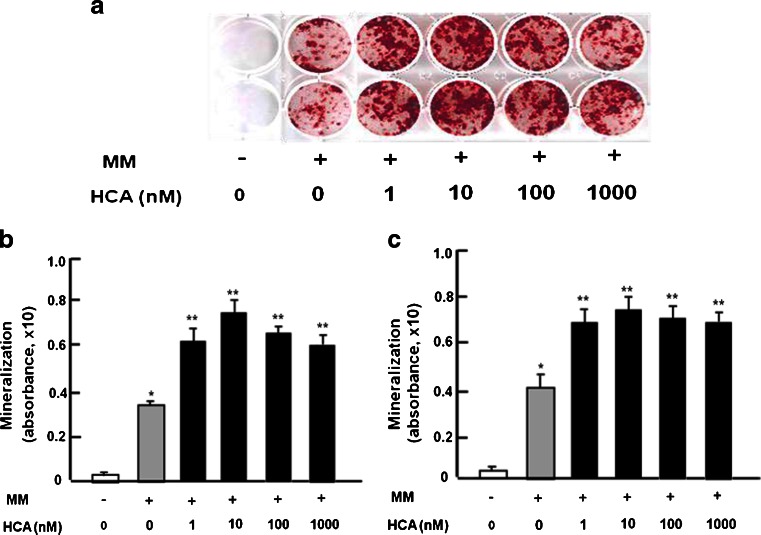
HCA stimulates osteoblastogenesis and mineralization in mouse bone culture in vitro. Bone marrow cells were cultured in 12-well plates in DMEM (10 % FBS and 1 % P/S) and mineralization medium (*MM*) containing ascorbic acid (100 ng/ml) and 4 mM β-glycerophosphate in the presence of either vehicle or HCA (1–1000 nM). **a** Cells were cultured for 21 days and the medium was changed every 3 days. After culture, cells were stained with Alizarin red stain. **b** For quantitation, 10 % cetylpyridinium chloride solution was added to each well to elute the Alizarin red dye. After complete elution, the absorbance of the eluted solution was measured at 570 nm on a microtiter plate reader. **c** Cells were cultured for 7 days in the presence of HCA (1–1000 nM) or its absence and then for an additional 14 days without HCA. Quantitation was performed as in **b** (*+* presence of reagents, *−* absence of reagents). Data are expressed as means ± SD of 8 replicate samples per data set. *P < 0.001 versus no treatment, **P < 0.001 versus MM-stimulated only (*gray bar*). One way analysis of variance (ANOVA), Tukey-Kramer post test

To determine the effects of HCA on osteoblastogenesis and mineralization, mouse bone marrow cells were cultured in medium containing either vehicle or mineralization medium in the presence of HCA (1–1000 nM) or its absence for 21 days. Culture with HCA stimulated osteoblastogenesis and mineralization (Fig. 1a, b). Such an effect was also seen when bone marrow cells were cultured in the presence of HCA for 7 days, after which the medium was replaced with medium without HCA and the cells were cultured for an additional 14 days (Fig. 1c). Thus, HCA probably stimulated the differentiation of osteoblasts from bone mesenchymal stem cells.

### HCA suppresses adipogenesis in bone marrow cells

**Fig. 2:**
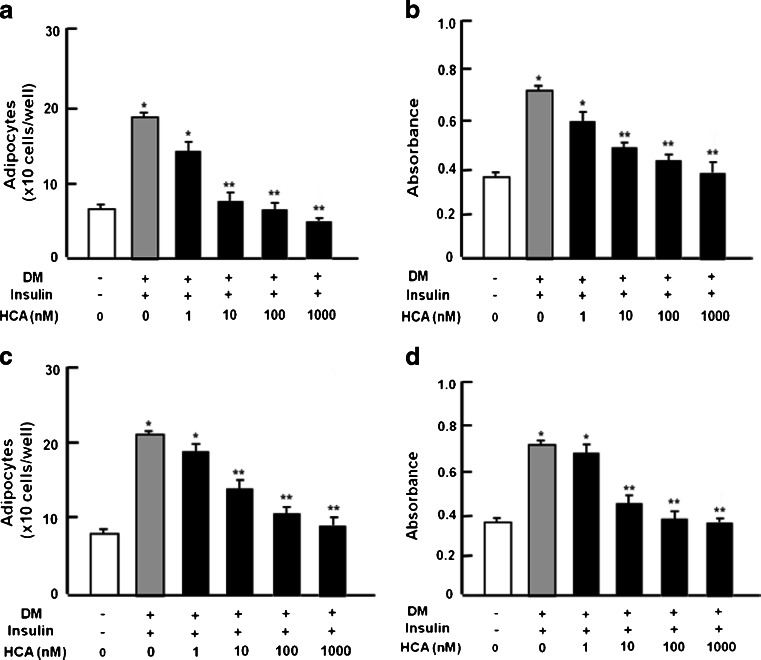
HCA suppresses adipogenesis in bone marrow culture in vitro. **a** Bone marrow cells (1 × 10^6^ cells/well per 1 ml in 12-well plates) were cultured for 48 h in DMEM (10 % FBS and 1 % P/S) containing either vehicle or HCA (1–1000 nM) in the presence (*+*) or absence (*−*) of differentiation medium (*DM*). The medium was replaced with α-MEM (10 % FBS and 1 % P/S) containing insulin (10 μg/ml medium) without dexamethasone and 3-isobutyl-1-methylxanthine (IBMX) and the cells were cultured in the presence of HCA (1–1000 nM) or its absence for 4 days. **c** Bone marrow cells were cultured in DM for 48 h with HCA (1–1000 nM) or without HCA and then the cells were cultured in medium containing insulin (10 μg/ml medium) for 4 days without HCA. After culture, the medium was removed and adipocytes were stained with Oil Red O. Adipocytes were counted by light microscopy (**a**, **c**). For quantification, the dye was extracted with isopropanol and the absorbance (490 nm) was read by using a Spectra Count microplate photometer (**b**, **d**). Data are expressed as means ± SD of 8 replicate samples per data set. *P < 0.001 versus no treatment; **P < 0.001 versus DM-stimulated only (*gray bar*). One way ANOVA, Tukey-Kramer post test

To determine the effects of HCA on adipogenesis, mouse bone marrow cells were cultured in medium containing vehicle or in DM in the presence of HCA (1–1000 nM) or its absence for 6 days. Culture with HCA suppressed adipogenesis (Fig. 2a, b). Such an effect was also seen when bone marrow cells were cultured in the presence of HCA for 2 days, after which the medium was replaced with the medium without HCA and the cells were cultured for an additional 4 days (Fig. 2c, d). Thus, culture with HCA suppressed adipogenesis in bone marrow cell culture.

### HCA suppresses adipogenesis in 3T3-L1 preadipocytes

**Fig. 3:**
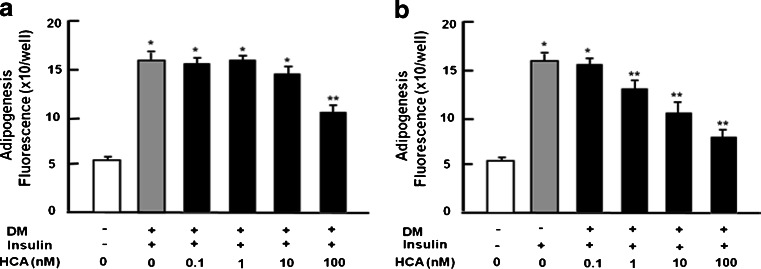
HCA suppresses adipogenesis in 3T3-L1 preadipocytes. **a** Subconfluent preadipocytes were stimulated with differentiation medium (*DM*) containing either vehicle or 0.5 mM IBMX and 1 μM dexamethasone for 2 days in the presence (*+*) of insulin (5 μg/ml medium) without HCA. Once mature adipocytes were seen, DM was replaced with medium containing insulin (5 μg/ml) in the absence (*−*) or presence (*+*) of HCA (1–100 nM) for 2 days (**a**, **b**). After such culture, DM was then replaced with medium containing insulin (5 μg/ml) without HCA (**a**) or with HCA (1–100 nM; **b**) for 2 days. After culture, lipid content was measured. Data are expressed as means ± SD of 8 replicate samples per data set. *P < 0.001 versus no treatment; **P < 0.001 versus DM-stimulated only (*gray bar*). One way ANOVA, Tukey-Kramer post test

To determine the effect of HCA on adipogenesis in preadipocytes, mouse 3T3-L1 preadipocytes were cultured in DM for 2 days with HCA (1–100 nM) or without HCA. The medium was then replaced with DMEM containing insulin without HCA (Fig. 3a). Adipogenesis was significantly decreased (Fig. 3a). In the second experiment, preadipocytes were cultured for 2 days in DM with HCA (1–100 nM) or without HCA followed by an additional 2 days in DMEM containing insulin and HCA (1–100 nM; Fig. 3b). HCA (10 and 100 nM) potently suppressed adipogenesis (Fig. 3b). These results indicated that longer culture with HCA had a potent suppressive effect in adipogenesis in the presence of insulin in vitro. HCA might suppress lipid production and/or stimulate lipid degradation in adipocytes.

HCA did not have a significant effect on cell viability. Subconfluent 3T3-L1 preadipocytes were cultured for 2 days in DM containing IBMX, dexamethasone and insulin in the presence of either the vehicle or HCA (0.1–100 nM) or their absence and were then cultured for 2 days in medium containing insulin in the presence of either the vehicle or HCA (0.1–100 nM) or their absence. The cell viability of mature adipocytes was not significantly changed after culture with HCA (data not shown), indicating that culture with HCA did not cause cell death.

### HCA suppresses insulin-stimulated lipogenesis in mature 3T3-L1 adipocytes

**Fig. 4:**
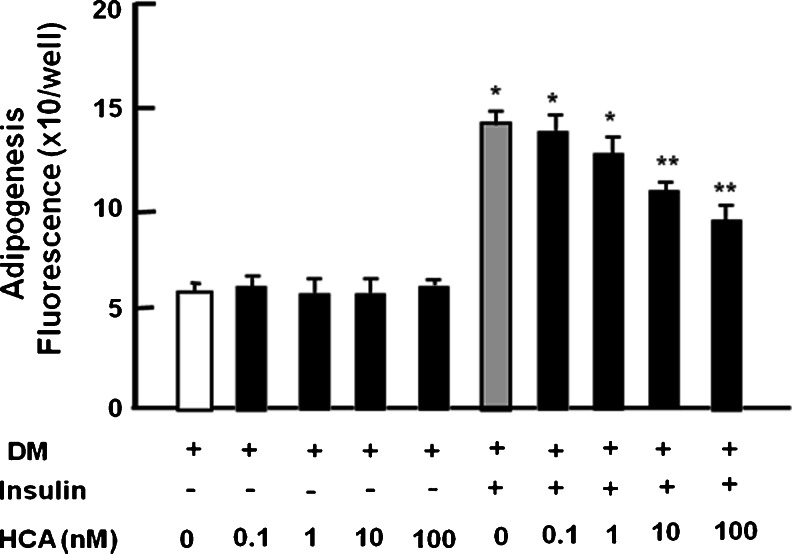
HCA suppresses insulin-stimulated lipid accumulation in 3T3-L1 mature adipocytes. Subconfluent preadipocytes were cultured in differentiation medium (*DM*) containing 0.5 mM IBMX and 1 μM dexamethasone for 2 days without HCA and then DM was replaced with medium containing either vehicle or insulin (5 μg/ml) in the presence (*+*) of HCA (0.1–100 nM) or absence (*−*) of HCA and the cells were cultured for an additional 2 days. After culture, lipid content was measured. Data are expressed as means ± SD of 8 replicate samples per data set. *P < 0.001 versus no treatment (*white bar*). One way ANOVA, Tukey-Kramer post test

**Fig. 5:**
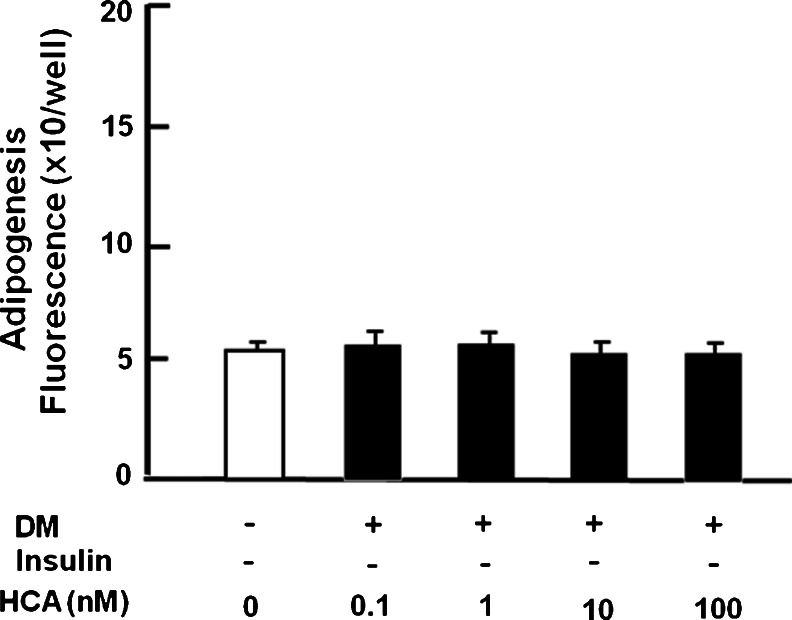
HCA does not have an effect on lipid content in 3T3-L1 adipocytes cultured in the absence (*−*) of insulin. Subconfluent preadipocytes were cultured in differentiation medium (*DM*) containing either vehicle or 0.5 mM IBMX and 1 μM dexamethasone for 2 days in the absence of insulin. DM was then replaced with medium containing either vehicle or HCA (0.1–100 nM) in the absence of insulin for 2 days. After culture, lipid content was measured (*+* presence). Data are expressed as means ± SD of 8 replicate samples per data set. No significant differences were seen. One way ANOVA, Tukey-Kramer post test

To characterize the effect of HCA on the insulin-stimulated lipid accumulation, 3T3-L1 preadipocytes were cultured for 2 days in DM without HCA followed by 2 days in DMEM with HCA (0.1 to 100 nM) or without HCA in the presence or absence of insulin. Culture with HCA did not cause a significant change in lipid content in mature adipocytes cultured in the absence of insulin (Fig. 4). This result indicated that HCA did not have an effect on insulin-independent lipid accumulation in mature adipocytes. However, HCA (10 and 100 nM) had a potent suppressive effect on lipogenesis in the presence of insulin (Fig. 4). Moreover, to determine whether HCA had an effect on lipolysis, undifferentiated preadipocytes were cultured in DM without insulin addition for 2 days and differentiated mature adipocytes were cultured in medium containing HCA (0.1–100 nM) in the absence of insulin for 2 days. Culture with HCA did not cause a significant change in lipid accumulation in adipocytes cultured in the absence of insulin (Fig. 5), suggesting that HCA did not have an effect on lipolysis in adipocytes.

### Effect of signaling inhibitors on HCA-induced suppression of adipogenesis in preadipocytes cultured with insulin

**Fig. 6:**
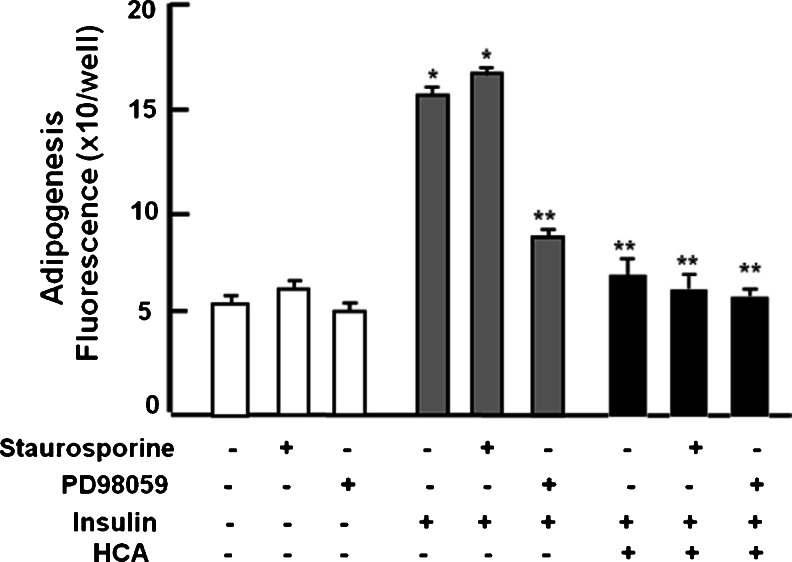
Suppressive effects of HCA on adipogenesis in 3T3-L1 preadipocytes is absent in the presence of PD98059. Subconfluent preadipocytes were stimulated with differentiation medium (*DM*) containing 0.5 mM IBMX plus 1 μM dexamethasone for 2 days in the presence (*+*) of insulin (5 μg/ml medium) or absence (*−*) of insulin with staurosporine (0.1 μM), PD98059 (1 μM), or HCA (100 nM) or without these factors. DM was then replaced with medium containing either vehicle or insulin (5 μg/ml) with staurosporine (0.1 μM), PD98059 (1 μM), or HCA (100 nM) or without these factors for 2 days. After culture, lipid content was measured. Data are expressed as means ± SD of 8 replicate samples per data set. *P < 0.001 versus DM-stimulated only (*white bar*); **P < 0.001 versus insulin-stimulated only (*gray bar*). One way ANOVA, Tukey-Kramer post test

To determine whether suppressive effects of HCA on insulin-stimulated adipogenesis are mediated through insulin signaling, mature adipocytes were cultured in the presence of staurosporine, an inhibitor of general protein kinases including protein kinase C (Chen et al. 2009), or PD98059, an inhibitor of MAPK, which is related to the extracellular-signal-related kinase (ERK) signaling pathway (Liu et al. 2012) (Fig. 6). Staurosporine or PD98059 alone did not have an effect on lipid content in adipocytes cultured without insulin. In the absence of HCA, PD98059 suppressed insulin-stimulated lipid accumulation in adipocytes but no additional suppression occurred when HCA (100 nM) was added to the medium. In the absence of HCA, staurosporine had no effect on insulin-stimulated adipogenesis but adipogenesis was suppressed when HCA (100 nM) was added. Thus, the suppressive effects of HCA on insulin-stimulated lipogenesis might involve the inhibition of the insulin-activated MAPK/ERK signaling pathway in adipocytes.

## Discussion

Osteoporosis and obesity are currently major health problems worldwide (Weitzmann and Pacifici 2006; Rosen and Bouxsein 2006) and one of their shared features is that osteoblasts and adipocytes differentiate from common precursor cells, namely mesenchymal stem cells, in the bone marrow. In previous experiments, HCA has been shown to stimulate osteoblastogenesis in preosteoblastic MC3T3-E1 cells (Yamaguchi et al. 2008a) and to suppress osteoclastogenesis in mouse bone marrow cells in vitro (Lai and Yamaguchi 2007a). Oral administration of HCA has been demonstrated to have an anabolic effect on bone in vivo (Lai and Yamaguchi 2006b, 2007b; Yamaguchi et al. 2008b). In this study, HCA has been found to have stimulatory effects on osteoblastogenesis and mineralization in mouse bone marrow cell culture in vitro, supporting findings that the compound has stimulatory effects on osteoblast differentiation and mineralization in preosteoclastic MC3T3-E1 cells in vitro (Yamaguchi et al. 2008a). In addition, HCA has been found to have suppressive effects on adipogenesis in mouse bone marrow cell culture in vitro. Osteoblasts and adipocytes differentiate from bone marrow mesenchymal stem cells (Muruganandan et al. 2009). The stimulatory effect of HCA on osteoblastogenesis and mineralization and its suppression of adipogenesis in bone marrow culture have also observed at earlier stages of culture during the differentiation of mesenchymal stem cells to preosteoblasts and preadipocytes. HCA might stimulate the process of differentiation to osteoblasts and suppress the process of differentiation of adipocytes from bone marrow mesenchymal stem cells. However, whether HCA has a direct effect on mesenchymal stem cells remains to be elucidated.

We also examined whether HCA has suppressive effects on adipogenesis during the process of differentiation from preadipocytes to mature adipocytes. Culture with HCA has been found to suppress adipogenesis in 3T3-L1 preadipocytes. HCA does not have an effect on the cell viability of adipocytes, indicating that the suppressive effects of HCA on adipogenesis do not result from cell toxicity and apoptotic cell death in preadipocytes and mature adipocytes. In addition, HCA does not stimulate lipolysis in mature adipocytes. HCA might thus suppress adipogenesis through other mechanisms but not via the stimulation of cell apoptosis and cell proliferation of adipocytes and lipolysis in mature adipocytes.

HCA suppresses adipogenesis in the differentiation process from preadipocytes to mature adipocytes in the presence of insulin. HCA might thus suppress adipogenesis during the process of differentiation from preadipocytes to mature adipocytes via a mechanism that is related to insulin action.

The activation of the MAPK/ERK signaling pathway during adipogenesis enhances the activity of factors that regulate the expression of both CCAAT/enhancer-binding protein (C/EBP) alpha and PPARγ (Prusty et al. 2002). Activation of the MAPK/ERK signaling pathway promotes PPARγ expression and phosphorylation and subsequently enhances the adipogenic differentiation of mesenchymal stem cells (Wu et al. 2010). The phosphorylation of Akt and ERK in 3T3-L1 preadipocytes functions as downstream signaling of insulin (Wu et al. 2010; Kato et al. 2007). PD98059 is an inhibitor of the MAPK/ERK signaling pathway (Kato et al. 2007). Culture with HCA suppresses insulin-stimulated adipogenesis in 3T3-L1 preadipocytes and mature adipocytes. Such an effect is not seen in culture with PD98059, which inhibits insulin-stimulated adipogenesis in adipocytes. HCA might thus suppress insulin-stimulated adipogenesis that is mediated through the MAPK/ERK signaling pathway. We speculate that HCA directly inhibits the activity of MAPK/ERK. The molecular mechanism by which HCA suppresses insulin-stimulated lipogenesis in adipocytes, however, remains to be elucidated.

In conclusion, this study demonstrates that HCA has stimulatory effects on osteoblastogenesis and mineralization and suppressive effects on adipogenesis in mouse bone marrow cell culture in vitro and that it has suppressive effects on insulin-stimulated adipogenesis in preadipocytes. Adipocyte hypertrophy and hyperplasia are important processes in the development of obesity, which induces osteoporosis. The flavonoid HCA might therefore have preventive effects on osteoporosis and obesity.
